# Satisfaction of Iranian Medical Universities’ faculty members towards holding Shahid Motahari Annual Educational Festival

**Published:** 2015-10

**Authors:** SEYYED NASROLLAH HOSSEINI, ANOSHIRAVAN MOHSENI BAND PEY, SEYYED ALI HOSSEINI, BEHZAD KARAMI MATIN, MEHDI MIRZAEI ALAVIJEH, FARZAD JALILIAN

**Affiliations:** 1 Ministry of Health and Medical Education, Tehran, Iran;; 2 Department of Environmental Health, Health School, Shahid Beheshti University of Medical Sciences, Tehran, Iran;; 3 Department of Occupational Therapy, University of Social Welfare and Rehabilitation Sciences, Tehran, Iran;; 4 Department of Public Health, Health School, Kermanshah University of Medical Sciences, Kermanshah, Iran

**Keywords:** Satisfaction, Attitudes, Education, Evaluation, Faculty members

## Abstract

**Introduction:**

Shahid Motahari Annual Educational Festival aims to improve the quality of medical education in the Islamic Republic of Iran, and has held since 2008. The present study was performed to determine the satisfaction level of Iranian medical universities’ faculty members about holding Shahid Motahari Annual Educational Festival during the past six years, from 2008 to 2014.

**Methods:**

This cross-sectional study was conducted on 473 faculty members (FMs) including deputies and educational administrators, managers, and faculty members of medical education development centers, members of scientific committees, and faculty members who participated in Shahid Motahari Festival from 42 medical sciences universities in Iran. Data collection instruments were two reliable and valid questionnaires on the background and also participants’ satisfaction towards Shahid Motahari Educational Festival. Data were analyzed using SPSS Software, version 14.

**Results:**

Among all participants, 30 FMs (6.3%) were educational deputies, 36 FMs (7.6%) managers of medical education development centers, 226 FMs (56.2%) members of scientific committees, 29 FMs (6.1%) members of the national committees, 343 FMs (27.5%) attendees, and 264 FMs (55.8%) had participated for retraining. The total satisfaction level of the participants was 73.3% which shows a good satisfaction level.

**Conclusion:**

The results identified the main important strength points such as “proposals’ review process at the country level” and weakness points such as “organizing the festival”.

## Introduction

Shahid Motahari Annual Educational Festival was held on May 2009 for the first time. The festival was conducted in two levels. First, each educational faculty presented at most two proposals about educational processes to compete in the festival after receiving accepting by the reviewers. At this level, the selected proposals were evaluated by the festival’s committee regarding the standards and criteria of the ministry of health and medical education and then each proposal received a score. Finally, according to the given scores, proposals were ranked and the highest score was known as the top one in each medical university. After choosing the best proposals in each medical university, they entered the country level competition where the proposals are evaluated and ranked by the national scientific committee in order to choose the best one (1-3).

This festival has been held annually in honor of Martyr Motahari. The first festival was held in May 2009 in Shahid Beheshti Medical Sciences University with 343 proposals, and finally 35 proposals were selected. The second festival started in November 2009 and the proposals were selected by the universities’ scientific committees, and then the proposals were sent to the festival for evaluation. Then, the festival was held in May, 2010 in Shiraz University of Medical Sciences, and 35 proposals were selected among 303 proposals from 41 universities (4).

The third festival was announced in July 2011 to focus on four aspects of educational programs, namely “innovative educational methods”, “new evaluation approaches”, “educational management and leadership”, and “consultant and guidance”, and was held in Iran Medical Science University. 363 proposals were sent to the festival and 35 proposals were selected by the referees (4).

The title “medical science education and health horizon in comprehensive scientific map” was selected for the forth festival in Mashhad in 2012. This festival started to work considering the guidelines according to scientific issues such as educational software progress, ethnicity and moral justice, and four above-mentioned aspects as well. 38 proposals were selected in different aspects all over the country (4).

The fifth festival was held in Mazandaran University in 2012, where 552 proposals were sent to the festival and 59 of them were selected. This festival included some changes in order to improve the quality (4).

To enhance the quality of the festival and for the sake of unity in judging the proposals at the university and country levels, the sixth festival invited the elite and interested university professors for the central scientific committee members and held different workshops for introducing some members of the university and country scientific committees (4). The title “dynamic medical education and intelligent movement in national production and self-efficiency” was chosen for the sixth festival. Shahid Beheshti University of Medical Sciences and Iran University of Medical Sciences cooperated to hold the festival in the conference hall of Iran University of Medical Sciences. Table 1 compares the trend of sending proposals to the six mentioned festivals, from 2009 to 2014.

**Table1:** Comparison of the trend of proposals to the six mentioned festivals from 2009 to 2014

	**Total sent processes**	**Investigated processes**	**Selected processes**
** 1^st^ year **	343	343	35
** 2^nd^ year **	303	303	35
** 3^rd^ year **	363	307	35
** 4^th^ year **	378	360	38
** 5^th^ year **	552	544	59
** 6^th^ year **	524	512	42

Accordingly, considering six years of holding this festival and introducing the medical science faculties around the country as the contributors and referees of the festival, the festival’s committee suggested the present study to determine the satisfaction level of the faculty members about holding Shahid Motahari festival in the past six years which was conducted in 2014. 

## Methods

The present study is a cross-sectional descriptive analytical study and the research population consisted of educational presidents, managers, faculty members of medical education development centers, scientific committee members, and all other faculty members attending Shahid Motahari Festival from all medical universities of Iran. To do this study, we distributed the questionnaires among 473 faculty members from 42 medical science universities.

To collect the data, two questionnaires were developed by the researchers, including general information and satisfaction questionnaire on the process of holding Shahid Motahari Educational Festivals during the past 6 years. The questionnaires were validated by the expert opinions and the reliability was estimated through a pilot study and using Cronbach’s Alpha which was .73; it shows an acceptable level of reliability. 

General information questionnaire included questions such as the name of the university, participant’s official rank (educational president, chairman, faculty members of medical educational study and development center, faculty members of different universities), type of participation in the festival (member of university referee committee, member of referee committee at the country level), background of attending the festival, and cooperation with educational proposals presented in the festival.

Satisfaction questionnaire included six statements (which are mentioned in the results section). 5-point Likert scale (from satisfied, fairly satisfied, no idea, fairly not satisfied, not satisfied) was applied for scoring the items from 5 to 1, respectively; it indicates at least 6 and at most 30 scores for the satisfaction level related to the past six festivals.

To rank the scores numerically, we divided the total scores by 3 and coded tem as weak, average, and good.

Data were processed using SPSS software version 14 and analyzed using descriptive (distribution, mean, and standard deviation) and inferential statistics (Pearson coefficient correlation, etc.). The significance level was considered 0.05. 

## Results

The mean and the standard deviation of the satisfaction level were 21.99 and 4.76, respectively. The participants gained 73.3% of the total score which shows an acceptable satisfaction level about the festival (Table 1). Among the six given statements, “practicality of the festival” and “review process of the proposals at the country level” gained the highest and the lowest scores, respectively (Table 2).

**Table 2:** Frequency of filed variables of participants in the study

**Variables**		**Num**	**Percent (%)**
**Participant position**	Educational president	30	6.3
	EDC CD manager	36	7.5
	EDC member	83	17.5
	Participant	295	62.4
	No answer	29	6.1
**Participants’ role in festival**	University committee	266	56.2
	Country committee	29	6.1
	No answer	178	37.6
**Participants experience of attending the festival**	Yes	343	27.5
	No	87	18.4
	No answer	43	9.1
**Cooperation in product of premier country process**	Yes	264	55.8
	No	143	30.2
	No answer	66	14

Faculty members who participated at the country level reported more satisfaction about “the practicality of the festival” (p=0.019) and “review process of proposals at the country level” (p=0.036) (Table 3).

**Table 3:** Mean, standard deviation and satisfactory field

**No.**	**Satisfaction **	**Mean± SD**
**1**	Festival secretariat function	3.85±0.98
**2**	Judgment of processes and products at university level	3.85±1.00
**3**	Previous holdings of the festival	3.78±0.91
**4**	Quantitative progress of the festival	3.65±1.00
**5**	Qualitative progress of the festival	3.45±1.02
**6**	Judgment of processes and products at country level	3.43±1.06

In addition, faculty members who had the experience of attending the festival expressed higher satisfaction about “the practicality of the festival” (p=0.003) and “festival quality progress” (p=0.013) (Table 4).

**Table 4:** Experience of attending country premier process based on festival secretariat function, judging processes and products at country level and festival quantity progress

**Variables **		**Num **	**Mean± SD**	**Standard mean error**	**p**
**Participation in producing premier country process**	Yes	256	3.98±0.90	0.056	0.019
	No	134	3.65±0.94	0.081	
**Attending the festival**	Yes	336	3.86±0.90	0.049	0.003
	No	77	3.50±0.94	0.108	
**Judgment of processes and products at country level**					
**Participation in producing premier country process **	Yes	254	3.57±1.04	0.065	0.036
	No	133	3.38±1.00	0.087	
**Festival quantitative progress**					
**Attending the festival**	Yes	334	3.47±1.00	0.054	0.013
	No	77	3.42±0.95	0.108	

## Discussion

According to the results, faculty members reported proper satisfaction level about holding Shahid Motahari Annual Educational Festival. Satisfaction of faculty members about different aspects of the festival is of great importance for improving the festival in different aspects.

In this regard, Ranjbar and Vahidshahi (2006) introduced faculty members as key components of the educational systems and universities who play an important role in improvement and development of educational system (5). In their qualitative research on the expectations of faculty members of medical universities, Changiz *et al.* (2013) indicated that faculty members’ satisfaction on offering educational services can be an index for investigating proper functions of medical studies and developing educational centers (6). Thus, it is important to consider the satisfaction level of faculty members on holding and expanding the festivals from different points of views. If they express their satisfaction about holding the festival in different aspects, it will be more rational to continue the festival and apply useful ideas in order to improve it. By the same token, Danesh Pajooh *et al. *(2005) studied the faculty members of Shiraz University of Medical Sciences and concluded that supporting faculties by the managers and university staff could decrease psychological pressure (7). The present study suggests the satisfaction of faculty members as a supportive factor for holding educational festivals.

Despite the acceptable satisfaction level about the festival among the faculty members, the present study aimed to investigate the strengths and weaknesses in order to enhance the quality of the festival. Reviewing the related literature indicated that there was no similar study in this aspect. 

In the present study, “practicality of the festival” received the highest score from the faculty members. Accordingly, the study of Hosseinian et al*.* (2000) on the faculty members of Hamedan University of Medical Sciences, it was suggested that such factors as university management and good relationships among them were the external motivational factors for the faculty members and play very important roles (8, 9). Furthermore, Hamidi *et al.* (2012) showed that supervising the organizations along with creating a good working environment and removing economical problems among the staff of teaching hospitals could affect their satisfaction levels (10). Considering investigations on the low satisfaction levels, the present study could be helpful to reach festival goals easier and faster. It could be concluded that good level of satisfaction among the participants can make stronger relationships among the participants and the organizers of the festival. In addition, satisfactions about the “judgment procedure” at university level and previous festivals which were directly administered by university faculties suggested proper policies to achieve the goals of the festival.

“Festival quantitative progress” item which gained the average score showed that participants were satisfied with the increasing number of proposals. According to Table 1, it is worth mentioning that some changes were applied in the festival from the fifth festival held in 2011, for example changing the scoring lists by the referees, developing more fields, making proposal providers unanimous for the referees, and assigning two phases of review at the university level so that the proposals be studied by three referees in the first phase and those who gain at least two positive votes be judged by three other referees in the second phase). Thus, the best proposal will be ranked based on the highest score in each field (9).

In order to improve the quality of the festival at university and country level, the feedbacks were collected by the festival organizers. Elite and interested faculty members of type II and III universities were invited as the members of central scientific committee and workshops were administered by university faculty members. The title “dynamic medical education and intelligent movement in national production and self-efficiency” was selected in the sixth festival. It was held in Razi conference hall of Iran University of Medical Sciences in cooperation with Shahid Beheshti University of Medical Sciences (9).

The least satisfaction level in this study was related to the “review of the proposals at the country level”. It is worth mentioning that recognizing the reasons of this dissatisfaction needs deeper studies in order to find the solutions for gaining higher levels of satisfaction and preventing the related problems, and finally to reach the ultimate goals of the festival. 

In some other studies, recognizing and removing the obstacles in achieving the goals were investigated. Mazloomy *et al.* (2013) indicated that removing the obstacles among students like motivation barriers by university staff and also organizational obstacles and finding statistical methodologies could increase the motivation (9). Ahmadinejad *et al.* (2002) studied the students’ satisfaction about clinical practices and showed that clinical training and theoretical education gained higher satisfaction levels, respectively. Three factors were reported important in their study: the number of the trainers and instructors, knowledge about epidemic sicknesses, and regulated educational plans. These factors were considered effective on the satisfaction of the students in all educational directions (11). Majdzadeh *et al.* (2008) suggested that most participants were satisfied with faculty members’ promotion, investigation and acceptance of research proposals, and attending short-term training courses; however, they were mostly not satisfied with study opportunities (12). Shirdel (2006) mentioned that more than half of the faculty members liked the availability of the required information in medical sciences universities (13). Mehrabian *et al.* (2012) also showed that faculty members of Gilan University of Medical Sciences were satisfied with non-electronic library of the university; however, they asked for electronic sources (14). In a study by Zolfaghari *et al.* (2009) it was reported that the majority of the students, and nursing and midwifery tutors in Tehran University of Medical Sciences were satisfied with using electronic training system (15). Ramezani *et al.* (2008) suggested relative satisfaction about using central library among faculty members of Kerman University of Medical Sciences (16).

The present study also identified the items with a high level of satisfaction which can be considered as the strong points for the next festivals. As it was shown in the results section, faculty members who participated in the country level showed higher levels of satisfaction about the “practicality of the festival” and “review process of the proposals at the country level”. Faculty members with the experience of attending the previous festivals also liked the “practicality of the festival” and “quantitative progress of the festival”; this suggests that more participation in the festival may lead to higher satisfaction of the faculty members. Other studies also reported the importance of participation of the faculty members. Mehdizade *et al.* (2012) studied the expectations of the faculty members who work in the nursing colleges in Tehran, Iran from the performance of the college dean; a quantitative method was used to show their exceptions such as planning to support faculty members, establishing justice, evaluating their performances, providing good working conditions, applying proper management methods, considering economic conditions, and improving educational and research qualities (17). Salehi *et al.* (2002) in their study on the cooperation of faculty members in managers’ decision making in Isfahan University of Medical Sciences showed that this process was more cooperative to faculty members than managers (18).

## Conclusion

Based on the results of the study, it seems crucial to improve the satisfaction level of the faculty members about holding and organizing Shahid Beheshti Annual Educational Festival to achieve the goals. However, the present study suggests more accurate studies to determine weaknesses and strengths of different items such as “review process of the proposals at country level” and “organizing the festival”. 
